# Editing of hnRNP K protein mRNA in colorectal adenocarcinoma and surrounding mucosa

**DOI:** 10.1038/sj.bjc.6602938

**Published:** 2006-01-10

**Authors:** K Klimek-Tomczak, M Mikula, A Dzwonek, A Paziewska, J Karczmarski, E Hennig, J M Bujnicki, P Brągoszewski, O Denisenko, K Bomsztyk, J Ostrowski

**Affiliations:** 1 Department of Gastroenterology, Medical Center for Postgraduate Education and Maria Skłodowska-Curie Memorial Cancer Center and Institute of Oncology, ul. Roentgena 5, Warsaw 02-781, Poland; 2 Laboratory of Bioinformatics and Protein Engineering, International Institute of Molecular and Cell Biology, Warsaw 02-109, Poland; 3 Department of Medicine, UW Medicine Lake Union, University of Washington, Seattle, WA 98109, USA

**Keywords:** hnRNP K protein, RNA editing, colorectal cancer, phosphorylation

## Abstract

The heterogeneous nuclear ribonucleoprotein K (hnRNP K) protein is an RNA-binding protein involved in many processes that compose gene expression. K protein is upregulated in the malignant processes and has been shown to modulate the expression of genes involved in mitogenic responses and tumorigenesis. To explore the possibility that there are alternative isoforms of K protein expressed in colon cancer, we amplified and sequenced K protein mRNA that was isolated from colorectal cancers as well as from normal tissues surrounding the tumours. Sequencing revealed a single G-to-A base substitution at position 274 that was found in tumours and surrounding mucosa, but not in individuals that had no colorectal tumour. This substitution most likely reflects an RNA editing event because it was not found in the corresponding genomic DNAs. Sequencing of RNA from normal colonic mucosa of patients with prior resection of colorectal cancer revealed only the wild-type K protein transcript, indicating that G274A isoform is tumour related. To our knowledge, this is the first example of an RNA editing event in cancer and its surrounding tissue, a finding that may offer a new diagnostic and treatment marker.

Colorectal cancer is one of the most frequent causes of morbidity and mortality from malignancy in Europe and North America (Didkowska *et al*, 2003; Jemal *et al*, 2005). Although improvements have been made in the surgical and nonsurgical therapies of colorectal cancer, the mortality remains high. A better understanding of the molecular basis of the disease is needed to develop new diagnostic tools and improve ways to prevent and treat colorectal cancer.

The heterogeneous nuclear ribonucleoprotein K (hnRNP K) protein is an ancient RNA-binding protein (Bomsztyk *et al*, 2004). Since it was originally identified as a component of the heterogeneous nuclear ribonucleoprotein particles, K protein has been also found in the cytoplasm and mitochondria where it is involved in multiple processes that compose gene expression (Bomsztyk *et al*, 2004). K protein contains three K homology (KH) domains that mediate RNA/DNA binding and the K-interactive (KI) region that recruits many protein partners (Bomsztyk *et al*, 1997, 2004).

K protein is upregulated in malignant cells (Dejgaard *et al*, 1994; Mandal *et al*, 2001; Ostrowski and Bomsztyk, 2003; Li *et al*, 2004), and it regulates the expression of genes involved in mitogenic responses, indicating that it has a direct role in cancerogenesis (Michelotti *et al*, 1995, 1996; Ostrowski and Bomsztyk, 2003; Ostrowski *et al*, 2004). We have shown that following mitogenic stimulation, K protein is recruited to gene loci known to be upregulated in malignant states, including c-myc (Ostrowski *et al*, 2003). This finding is consistent with the observed shift of K protein to the nuclei in neoplasms (Ostrowski and Bomsztyk, 2003).

As a result of alternative splicing, there are several known isoforms of K protein (Dejgaard *et al*, 1994; Makeyev and Liebhaber, 2002). To explore the possibility that there are isoforms of K protein enriched in cancer, we sequenced cDNA encoding K protein from colorectal adenocarcinoma and compared it to the cDNAs from the surrounding normal mucosa. Sequencing revealed novel G274A polymorphism found in mRNA, but not in the genomic DNA. Thus, this polymorphism most likely reflects mRNA editing event, which changes alanine (Ala) to threonine (Thr) at the end of KH1 domain. Beside colorectal adenocarcinoma, G274A isoform of K protein mRNA was also found in a few thyroid cancers, but not in normal tissues surrounding breast, thyroid or renal carcinomas, nor in normal or in inflammation-altered colonic mucosa from the patients with inflammatory bowel disorders. These data identify novel RNA editing phenomenon specific to colorectal carcinoma that may be involved in the processes of tumour formation and development and may serve as a diagnostic marker.

## MATERIALS AND METHODS

### Tissues

In all, 21 surgically resected colorectal tumours, 18 thyroid tumours (five follicular, four papillary and nine medullary carcinomas), 20 breast carcinomas and 10 clear-cell renal carcinomas used in this study were obtained at the Maria Skłodowska-Curie Memorial Cancer Center and Institute of Oncology (Warsaw, Poland). Fresh surgical specimens were immediately placed on ice and transported to the pathology laboratory. Following gross examination, samples of adjacent tissue and tumours were dissected and immediately frozen at −80°C. The presence of normal and tumour tissue was assessed by histological evaluation of tissue adjacent to the fragments used for RNA and DNA isolation.

In 10 patients who underwent surgical treatment (but no adjuvant chemo- or radiotherapy) for colorectal carcinomas, mucosal biopsies were taken colonoscopically from macroscopically normal mucosa adjacent to the anastomosis, 12 to 26 months after the surgery. Also, in four patients with Crohn's disease and in five with ulcerative colitis, mucosal biopsies were taken colonoscopically from macroscopically normal colonic mucosa and mucosa involved in inflammatory process. The presence of normal mucosa and colonic inflammation typical for the inflammatory bowel disorder was confirmed histologically.

The protocol of the study was approved by local ethics committees, and written informed consent was obtained from all patients.

### RNA analysis

**Figure 1:**
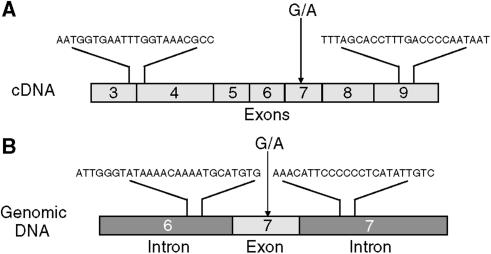
Position of the primers used for the amplification of hnRNP K cDNA (**A**) and genomic DNA (**B**) fragments.

Total RNA and genomic DNA tissue samples were purified using RNeasy Mini Kit and DNeasy Tissue Kit (Qiagen, Hilden, Germany), respectively. RNA was reverse transcribed using SuperScript II RT (GIBCO-BRL, Gaithersburg, MD, USA) and random hexamer primers in 20 *μ*l of volume as per the manufacturer's protocol. The full-length cDNA, a 453-bp fragment of cDNA or 301-bp fragment of genomic DNA encoding K protein were amplified using *Pfu Turbo* DNA polymerase and primers, as listed in the Table 1; primer location is shown in Figure 1.

The polymerase chain reaction (PCR)-amplified DNA fragments were T/A subcloned into pCR2.1 plasmid, and the resultant vectors were transformed into chemically competent TOP10 cells. Plasmid DNA was isolated from randomly chosen bacterial clones and sequenced using ABI Prism 377 automated DNA sequencing system (Applied Biosystems, Foster City, CA, USA).

Since G-to-A substitution at position 274 in K protein cDNA creates *Sca*1 restriction enzyme site (AGTACT), restriction fragment length polymorphism (Jenkins, 2004) was also used to screen cDNA fragments for the modification. Fragment (453 bp) of cDNA inserted into plasmid vector was amplified, the PCR product was purified with QIAquick PCR Purification Kit (Qiagen), digested with *Sca*I and analysed on 2.5% agarose gel.

### K protein phosphorylation *in vitro*

The wild-type and G274A-mutated cDNA of K protein were subcloned into pET-28(+) expression vector (Novagen, Madison, WI, USA), and the plasmids were transformed into *Escherichia coli* BL21 DE3 pLysS cells (Novagen). Bacterially expressed recombinant proteins were purified by affinity chromatography using Ni-NTA agarose (Qiagen) according to the manufacturer's protocol. The correct point-mutant product was confirmed by sequence analysis using liquid chromatography–electrospray mass spectrometry with collisional fragmentation (LC–ESI–MS–MS/MS).

Purified wild-type and mutated K proteins were phosphorylated *in vitro* by casein kinase-1 (CK1), casein kinase-2 (CK2) or their mixture in the buffer containing 25 mM Tris, pH=7.5, 200 mM NaCl, 0.1 mM ATP (with or without [*γ*-^32^P]-ATP) and 10 mM MgCl_2_ for 20 min at 30°C. Proteins phosphorylated in the presence of [*γ*-^32^P]-ATP were separated by sodium dodecyl sulphate–polyacrylamide gel electrophoresis and visualised using a phosphorimager. Proteins phosphorylated with cold ATP were digested with trypsin, and the resulting phosphopeptides were analysed by mass spectrometry. Peptide mixture was applied to nano-high-performance liquid chromatography RP-18 column, directly coupled to nano-Z-spray ion source of quadrapole time-of-flight electrospray mass spectrometer (Waters) working in the regimen of data-dependent MS to MS/MS switch. The output list of parent and daughter ions was submitted to database search using MASCOT (MatrixScience) program. Peptide identification and the presence of their covalent modifications were verified by inspection of parent mass fragmentation patterns using programs MassLynx (Waters) and ProteinProspector.

### Protein modelling

The three-dimensional (3D) structures of the KH1 domain of protein K and its mutant A92T, and of the KH2 domain were predicted by homology modelling using SWISS-MODEL (Peitsch, 1996), using the coordinates of the KH3 domain as the template (crystallographic structure 1 khm in Protein Data Bank). The structure of the entire K protein was modelled by joining the structures of the individual domains by the linker in an extended conformation. The preliminary model of KH1–RNA complex was obtained by copying the coordinates of the RNA molecule from the KH3–RNA structure obtained by NMR (Braddock *et al*, 2002) and optimising the interactions with the protein by the steepest descent method, as implemented in HyperChem 7.1 (Hypercube Inc., Gainesville FL, USA).

Putative phosphorylation sites in K protein were predicted using the NetPhos 2.0 server (http://www.cbs.dtu.dk/services /NetPhos/) (Blom *et al*, 1999). The 3D model of protein K was used to identify those potential phosphorylation sites, which are not buried in the protein core, and thus are likely to be modified in the native protein.

#### Statistical analysis

Differences between groups were analysed by the Mann–Whitney test and paired two-tailed *t*-test using Statistica PL software. A *P*-value of <0.05 was considered to be statistically significant.

## RESULTS

**Figure 2:**
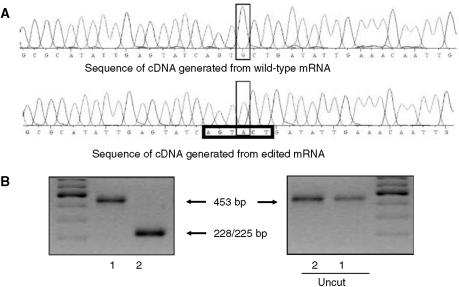
Analysis of K protein transcript. (**A**) Sequences of individually cloned cDNA fragments from wild-type and edited mRNA of K protein. *Sca*I restriction site is indicated by box. (**B**) Agarose gel electrophoresis of PCR-generated fragments from cDNA encoding K protein that were digested with (right) or without (left) *Sca*1 restriction enzyme. Fragments (453 bp) of K protein cDNA wild type (line 1) and with G274A polymorphism (line 2) were digested with *Sca*1.

Pilot sequencing of the full-length cDNA encoding K protein isolated from three pairs of colon adenocarcinoma and the normal mucosa surrounding the tumour revealed G274A polymorphism present in all samples (Accession number AY911506). To further explore this finding, cDNAs were generated from RNA isolated from several pairs of tissues (normal/pathological) that were taken from patients with colorectal cancer. A 453-bp fragment of K protein cDNA was amplified by PCR, T/A subcloned into plasmid vector and six to 14 bacterial clones from each transformation were randomly chosen for sequencing (Figure 2).

As shown in Table 2, G274A polymorphism was found in K protein cDNA generated from RNA that was isolated from 15 out of 21 (71%) colorectal cancers and from all normal mucosa that surrounded these colon tumours. The median percentage of K protein transcripts carrying G274A polymorphism within an individual tissue specimen were significantly higher in normal mucosa surrounding tumours than in corresponding colorectal carcinomas (54 *vs* 8% of all sequenced clones; *P*<0.001). One explanation for such a difference could be that tumour induces G274A RNA editing. Alternatively, normal mucosa in response to tumour may produce a signal for the RNA editing. The relative frequency of the clones with G274A polymorphism did not appear to correlate with any of the variables that were tested, such as sex, age, tumour location, size, type, differentiation, presence of metastases and stage according to Astler–Coller, Jass prognostic criteria. This observation suggests that G274A polymorphism is a good candidate to be used as a universal and specific marker for the diagnosis of colorectal adenocarcinoma.

The above studies suggested that the G274A polymorphism is related to colorectal adenocarcinoma. To test this relationship further, we sequenced K protein cDNA from normal mucosa taken colonoscopically from the sites of surgical anastomosis from 10 patients with prior resection of colorectal carcinoma, including two patients whose tissues were previously tested positive for the G274-A polymorphism in both cancer and surrounding mucosa. In all those patients, only the G274 wild-type isoform was found. Similarly, in nine patients with inflammatory bowel disease, sequencing analysis of biopsy specimens of the involved and unaffected mucosa revealed only the wild-type K protein mRNA. To test if the G274A polymorphism is specific to colorectal adenocarcinoma, we examined pairs of neoplastic and normal surrounding tissues from breast, kidney and thyroid. Except for two thyroid cancers, only the wild-genotype K protein RNA isoform was found in these specimens (Table 2). The fact that the G274A polymorphism was also found in thyroid cancers suggests that the RNA editing could be also induced by other tumours, although with much lower efficiency.

To exclude the possibility that the G274A polymorphism was generated by reverse transcriptase (RT)–PCR, we repeated the entire genotyping experiment in all colorectal patients. The second series of RNA extraction and sequencing confirmed the presence of previously determined polymorphism in both normal and cancer samples (data not shown). This indicates that G274A polymorphism was not introduced by PCR.

Next, we tested if G274A polymorphism is found in K protein gene. To do that, we analysed 78 samples of genomic DNA (Figure 1) obtained from tumour and matched surrounding normal tissue samples extracted from the same 21 patients with colorectal cancer and 18 patients with thyroid cancer. The PCR products were subcloned into plasmid vector, and five clones from each bacterial transformation were sequenced. Of 390 independent clones analysed, none contained the G274A polymorphism. Since there is only one K protein gene transcribed from 13 kb locus localised on long arm of chromosome 9, and K protein's pseudogenes have the same G274 base and are not expressed, we conclude that the G274A polymorphism most likely results from an RNA editing event.

**Figure 3:**
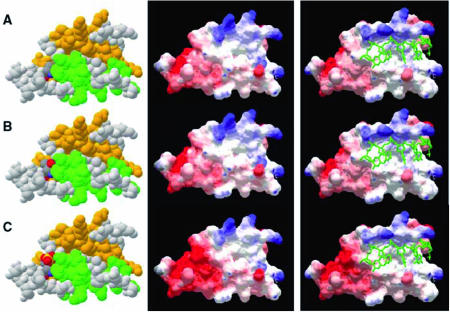
Structural modelling of wild-type, Thr92 and phospho-Thr92K protein isoforms. (**A**–**C**) model of the KH1 domain of the K protein, its A92T isoform and the A92T isoform phosphorylated at Thr92, respectively. The protein is shown in the space fill representation, with helices shown in orange, strands in green and loops in light grey. Residue 92 (Ala, Thr and phospho-Thr, respectively) is coloured according to the following scheme: C atoms: dark grey; O atoms: red; N atoms: blue; and P atoms: orange. (**D**–**F**) The Coulomb electrostatic potential mapped onto the surface of each variant of the KH1 domain and expressed as a colour range (from red, −6 kT, to blue, +6 kT*).* (**G–I**) Model of RNA interacting with KH1 domain of hnRNP K protein.

G274A polymorphism found in K protein mRNA substitutes Ala codon by Thr codon. One consequence of such amino-acid substitution within K protein could be a creation of new phosphorylation site. Reversible phosphorylation of proteins is a fundamental mechanism of the regulation of cellular functions. Analysis of K protein amino-acid sequence revealed several putative sites that meet the criteria for phosphorylation by serine–threonine kinases: calmodulin CII, CK1, CK2, glycogen synthase kinase 3, p34cdc2, p70S6 and protein kinase C (PKC). Indeed, K protein has been shown to be phosphorylated by several kinases, including Src, Lck, PKC*δ*, extracellular signal-regulated protein kinse, c-Jun NH2-terminal kinase and CK2, each phosphorylating one or more sites (Bomsztyk *et al*, 2004). Three-dimensional modelling of K protein indicates that Thr92, introduced as a result of G274A editing of mRNA, is located on the surface of the molecule (Figure 3; panels A–C), and thus may represent a new phosphorylation site for CK1. We tested this possibility next.

**Figure 4:**
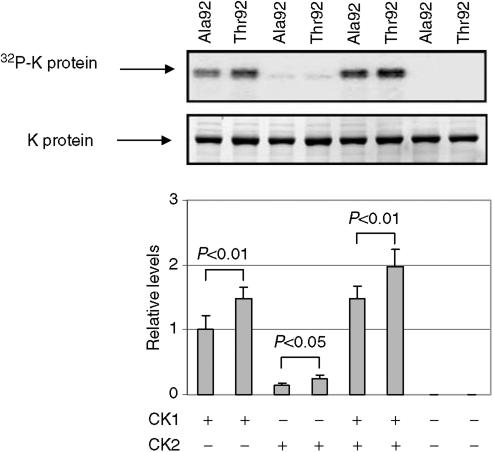
Phosphorylation of K protein and its mutant by casein kinases (CK). Recombinant wild-type and Thr92-mutated K proteins were phosphorylated using CK1 (CK1*δ*, rat, recombinant) or CK2 (CKII purified from rat liver) or both kinases. ^32^P-labelled proteins eluted from the beads were analysed by sodium dodecyl sulphate–polyacrylamide gel electrophoresis followed by autoradiography (upper panel) and densitometry of the Coomassie-stained band of the same gel (lower panel) using Molecular Imager FX Pro Plus (Bio-Rad). The ^32^P signals were normalised to Coomassie band intensities and expressed as relative levels (diagram). Results shown in the graph represent means±s.d. of three independent experiments.

As shown in Figure 4, CK1 effectively phosphorylates both the wild-type protein and A92T isoform, whereas phosphorylation mediated by CK2 was much weaker. However, the strongest signals were generated by the use of mixture of both enzymes CK1 and CK2 in phosphorylation reactions. The intensity of phosphorylation was significantly stronger for Ala92Thr-mutated protein than the wild-type K protein.

To prove that increased phosphorylation of mutated K protein reflects phosphorylation of Thr92, we used mass spectrometry analysis. The wild-type and mutated proteins phosphorylated by both CK1 and CK2 were digested with trypsin. The resulting peptides were analysed by LC–ESI–MS–MS/MS mass spectrometry. Amino-acid sequencing identified the 87- to 103-amino-acid wild-type K protein peptide (ILSIS**A**DIETIGEILKK) (bold A indicates Ala at position 92) and the mutated peptide (ILSIS**T**DIETIGEILKK) (bold T indicates substituted Thr at position 92) molecules with the masses 1843.07 and 1873.08 Da, respectively, as well as with 80 and 160 Da above the predicted masses. The addition of a phosphate group increases the mass of peptides by 80 Da. Thus, as shown in Table 3, the A92T isoform was much more efficiently phosphorylated by these kinases, an alteration that most likely reflects direct phosphorylation of Threonine 92.

In the wild-type peptide, there are three potential phosphorylation sites adjacent to Ala92, Ser89, Ser91 and Thr96, but only Thr96 fits the criteria for phosphorylation by CK2. Thus, the characteristic mass shift of 0.7% of the wild-type peptide molecules likely reflects P-Thr96. The Thr92 residue, introduced by RNA editing, also meets the criteria for phosphorylation by either CK1 or CK2. Therefore, 22.5% of mutated peptide molecules with added 80 Da might reflect P-Thr92, and other 2.5% of phosphopeptides are consistent with both P-Thr92 and P-Thr96. Thus, MS studies provide evidence that Thr92 residue is either itself phosphorylated or enhances phosphorylation of the neighbouring Ser/Thr residues.

K protein binding to RNA reflects synergistic interaction between three KH domains and cognate RNA where each KH domain binds one of three C-rich boxes (Paziewska *et al*, 2004). As shown in Figure 3, the structural changes resulting from the putative phosphorylation of Thr92 does not disrupt the 3D-fold of the KH domain (panels A–C). However, the phosphorylation can introduce significant negative charge on the protein surface (panels D–F), leading to alteration of K protein RNA-binding activity. Independent of whether Thr92 is directly phosphorylated or changes phosphorylation of other neighbouring residues, these observations indicate that A92T polymorphism may alter K protein function in response to extracellular signals that engage kinase cascades. The putative location of the RNA molecule (panels G–I) (based on solved structure of the KH3 single-stranded DNA complex; Braddock *et al*, 2002) is shown to visualise the region of protein–RNA interactions that may be influenced by the Ala to Thr substitution and by the phosphorylation. Although not shown in this figure, because of the lack of the appropriate modelling template, the extended 3′-end of the RNA molecule could directly interact with the modified region.

## DISCUSSION

RNA editing is a co- or post-transcriptional process (Gott and Emeson, 2000) that modifies the genetic information at the transcript level, either by insertion or deletion of nucleotides, or by base modification. Enzymatic base modification of RNA sequences was originally discovered in the mitochondrial RNA of trypanosomes (Turelli and Trono, 2005). In mammals, the two major types of RNA base modifications are associated with various physiological processes, including neurotransmission, lipid metabolism and adaptive immunity (Turelli and Trono, 2005). They include A-to-I and C-to-U editing (Gott and Emeson, 2000; Maas and Rich, 2000; Davidson, 2002).

RNA editing is mediated by RNA–protein complexes, which contain small guiding RNAs and several associated proteins (Decatur and Fournier, 2003). A-to-I RNA editing occurs on unspliced mRNA templates and is mediated by adenosine deaminases acting on double-stranded RNAs). The partially double-stranded RNA that involves exonic and intronic sequences or a hairpin structure is thought to be essential for this type of RNA editing (Maas *et al*, 2003). The primate cytidine deaminases family represents 12 enzymes (Turelli and Trono, 2005). Adenosine deaminases acting on double-stranded RNAs are responsible for site-specific RNA editing of both coding and noncoding region sequences (Yang *et al*, 2005). Adenosine deaminases acting on double-stranded RNA-mediated RNA editing occurs within coding or untranslated RNAs and appears to be widespread in human nervous system (Bhalla *et al*, 2004; Blow *et al*, 2004). Adenosine deaminases acting on double-stranded RNA-mediated RNA editing is essential for normal development and cell viability, and altered editing is associated with inflammation and several neurological disorders (Geslain and Ribas de Pouplana, 2004; Eisenberg *et al*, 2005).

Until now, a few C-to-U edits and single examples of purine-to-pyrimidine and pyrimidine-to-purine RNA edits were described within the open-reading frame of cellular and viral transcripts (Gott and Emeson, 2000; Maas and Rich, 2000; Gerber and Keller, 2001; Schaub and Keller, 2002; Decatur and Fournier, 2003; Vitali *et al*, 2005). Cytidine deamination was recognised as an important defence mechanism against retroviral infection, including human and simian immunodeficiency viruses, equine infectious anaemia virus and murine leukaemia virus (Turelli and Trono, 2005).

**Figure 5:**
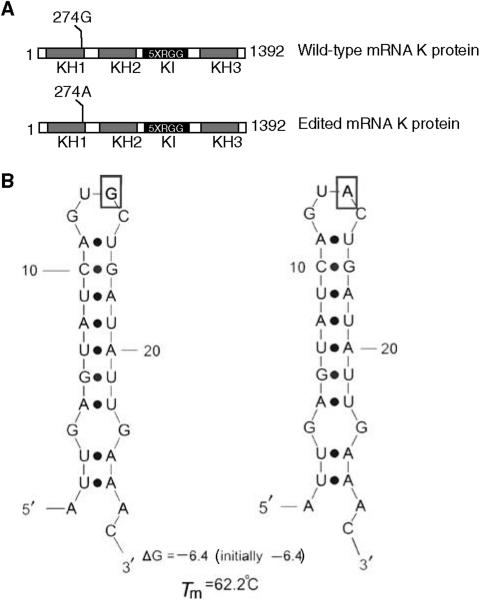
mRNA editing of G to A generates a novel K protein Thr92 isoform. (**A**) The G to A base substitution at 274 in K protein transcript changes Ala92 to Thr92 located at the end of the KH1 domain. KH domains are shown in green. (**B**) K protein RNA sequence analysis around the editing site reveals a hairpin conformation.

Based on the discrepancy between the mRNA (cDNA) and genomic sequences, we have identified G274A RNA editing of K protein transcript specific to adenocarcinomas of the colon. Similar G-to-A editing events were also described in mRNA of the human EAA5 (GluR7) receptor (Nutt *et al*, 1994) and in human immunovirus-1 mRNAs isolated from chronically infected H9 cells (Bourara *et al*, 2000). Mechanisms of G-to-A RNA editing are unknown. No consensus was detected in surrounding sequences of viral RNA that underwent G-to-A editing and there were no consistent secondary structures evident in these regions (Bourara *et al*, 2000). Nonetheless, analysis of K protein RNA sequence around the editing site revealed a potential hairpin conformation (Figure 5B). Whether this secondary structure guides G274A editing remains to be determined.

RNA editing that involves changes in mRNA sequences may create new start or stop codons, new open-reading frames or alteration in encoded amino acids. Modification of untranslated regions of a transcript can alter its stability, transport or translation, and intronic editing can influence pre-mRNA splicing. Although RNA editing may change genetic information that, in turn, may generate genetic instability in neoplasms, only few examples of such alterations were described in neoplasm. For example, altered expression of NAT1 (homologous the caboxyl-terminal translational initiation factor 4G) and NF1 (encoding neurofibromatosis type 1 protein) that resulted from C-to-U RNA editing was described in malignant tissues (Aasheim *et al*, 1994). In human carcinomas of the stomach, pancreas, large intestine and liver alterations in the expression of an RNA-specific cytidine deaminase, APOBEC-1, that is involved in RNA editing were also reported (Lee *et al*, 1998). Therefore, RNA editing found in malignant tissues may be a cause or a consequence of tumorigenesis.

Although the method used to estimate the frequency of the RNA editing events allowed only for a semiquantitative analysis, the polymorphism of K protein mRNA seems to be more common in the surrounding normal mucosa than in colorectal cancer. Since G274A polymorphism was absent in colon mucosa from patients who underwent a colon resection for colorectal cancer, or from patients with inflammatory bowel disease, or in neoplastic and normal surrounding tissues from breast, kidney and thyroid, we hypothesise that it may be a field defect in colorectal mucosa surrounding the cancer where tumour releases a signal for the RNA editing. It is also possible that G274A isoform of K protein is a defence reaction of normal tissues to the neighbouring tumour. Future experiments will show whether RNA editing of K protein contributes to the development of colorectal cancer.

As K protein is upregulated in tumour cells (Dejgaard *et al*, 1994; Mandal *et al*, 2001; Ostrowski and Bomsztyk, 2003; Li *et al*, 2004) and modulates the expression of genes involved in mitogenic responses (Michelotti *et al*, 1995, 1996; Mandal *et al*, 2001; Ostrowski *et al*, 2004), altering its properties and introduction of a new phosphorylation site could make K protein either more or less conducive for the cellular processes that lead to tumorigenic state. If so, identification of this novel tumour-associated K protein isoform may prove to be a useful diagnostic, prognostic and/or therapeutic indicator.

## Figures and Tables

**Table 1:** Primers used to amplify K protein cDNA

	**Primer sequence 5′ → 3′**	**Expected length of PCR product**	***T*_a_** **(°C)**
Full-length cDNA	GCTTCAGTTCTGCTCTGCAAGGAT-fwd	1619	57
	ACACCTCAAATGCAGAACACCTATGA-rev		
RFLP fragment	AATGGTGAATTTGGTAAACGCC-fwd	453	57
	TTTAGCACCTTTGACCCCAATAAT-rev		
Genomic fragment	ATTGGGTATAAAACAAAATGCATGTG-fwd	301	54
	AAACATTCCCCCCTCATATTGTC-rev		

PCR=polymerase chain reaction; RFLP=restriction fragment length polymorphism.

**Table 2:** Summary of G274A RNA polymorphism analysis

**Patient groups**	**Number of tissue pairs**	**Number of tissues with G274A RNA isoform Normal tissue/pathological tissue**	**Number of sequenced clones: total (*G274A RNA*) Normal tissue/pathological tissue**
Colon cancer	21	21/15	229 (***120***)/235 (***51***)
Colon cancer after surgical treatment	10	0/NA	80 (***0***)/NA
Crohn's disease or ulcerative colitis	9	0/0	54 (***0***)/54 (***0***)
Thyroid cancer	18	0/2	108 (***0***)/108 (***5***)
Breast cancer	20	0/0	120 (***0***)/120 (***0***)
Renal cancer	10	0/0	80 (***0***)/80 (***0***)

NA=not applicable.

**Table 3:** Summary of peptide (87–103 aa) counts derived from the wild-type and A92T-mutated K protein that were phosphorylated by CK1 and CK2 *in vitro* and analysed by mass spectrometry

	**ILSIS*A*DIETIGEILKK mass 1843.07 Da (% of total)**	**ILSIS*T*DIETIGEILKK mass 1873.08 Da (% of total)**
Total peptide counts^a^	1569 (100)	3360 (100)
Number of peptide molecules with the mass 80 Da above predicted	10 (0.7)	755 (22.5)
Number of peptide molecules with the mass 160 Da above predicted	ND	86 (2.5)

aa=amino acids; CK1=casein kinase 1; CK2=casein kinase 2; ND=not detected.

a Total peptide counts is the number of detected peptide molecules with the predicted mass as well as with the masses 80 and 160 Da above predicted. Italic A or T in peptides sequences indicates Ala or substituted thr at position 92, respectively.
